# Ligand-based drug design against Herpes Simplex Virus-1 capsid protein by modification of limonene through in silico approaches

**DOI:** 10.1038/s41598-024-59577-4

**Published:** 2024-04-29

**Authors:** Md. Rezaul Islam, Md. Shafiqul Islam Sovon, Ummy Amena, Miadur Rahman, Md. Eram Hosen, Ajoy Kumer, Mohammed Bourhia, Yousef A. Bin Jardan, Samir Ibenmoussa, Gezahign Fentahun Wondmie

**Affiliations:** 1 Department of Pharmacy, Faculty of Allied Health Sciences, Daffodil International University, Dhaka, Bangladesh 1207; 2 Department of Pharmacy, Faculty of Pharmacy, University of Dhaka, Dhaka, 1000 Bangladesh; 3 Department of Pharmacy, Faculty of Life & Earth Sciences, Jagannath University, Dhaka, Bangladesh; 4 Department of Pharmaceutical Sciences, North South University, Dhaka, 1219 Bangladesh; 5 Department of Genetic Engineering and Biotechnology, University of Rajshahi, Rajshahi, 6205 Bangladesh; 6 Department of Chemistry, College of Arts and Sciences, International University of Business Agriculture and Technology (IUBAT), Dhaka, 1216 Bangladesh; 7 Center for Global Health Research, Saveetha Institute of Medical and Technical Sciences in Saveetha Medical College and Hospital, Chennai, India; 8 Laboratory of Biotechnology and Natural Resources Valorization, Faculty of Sciences, Ibn Zohr University, 80060 Agadir, Morocco; 9 Department of Pharmaceutics, College of Pharmacy, King Saud University, P.O. Box 11451, Riyadh, Saudi Arabia; 10 Laboratory of Therapeutic and Organic Chemistry, Faculty of Pharmacy, University of Montpellier, 34000 Montpellier, France; 11 Department of Biology, Bahir Dar University, P.O. Box 79, Bahir Dar, Ethiopia

**Keywords:** Herpes Simplex Virus-1, Molecular docking, DFT, ADMET, Molecular dynamics simulation, Computational biology and bioinformatics, Drug discovery

## Abstract

The pharmacological effects of limonene, especially their derivatives, are currently at the forefront of research for drug development and discovery as well and structure-based drug design using huge chemical libraries are already widespread in the early stages of therapeutic and drug development. Here, various limonene derivatives are studied computationally for their potential utilization against the capsid protein of Herpes Simplex Virus-1. Firstly, limonene derivatives were designed by structural modification followed by conducting a molecular docking experiment against the capsid protein of Herpes Simplex Virus-1. In this research, the obtained molecular docking score exhibited better efficiency against the capsid protein of Herpes Simplex Virus-1 and hence we conducted further in silico investigation including molecular dynamic simulation, quantum calculation, and ADMET analysis. Molecular docking experiment has documented that Ligands 02 and 03 had much better binding affinities (− 7.4 kcal/mol and − 7.1 kcal/mol) to capsid protein of Herpes Simplex Virus-1 than Standard Acyclovir (− 6.5 kcal/mol). Upon further investigation, the binding affinities of primary limonene were observed to be slightly poor. But including the various functional groups also increases the affinities and capacity to prevent viral infection of the capsid protein of Herpes Simplex Virus-1. Then, the molecular dynamic simulation confirmed that the mentioned ligands might be stable during the formation of drug-protein complexes. Finally, the analysis of ADMET was essential in establishing them as safe and human-useable prospective chemicals. According to the present findings, limonene derivatives might be a promising candidate against the capsid protein of Herpes Simplex Virus-1 which ultimately inhibits Herpes Simplex Virus-induced encephalitis that causes interventions in brain inflammation. Our findings suggested further experimental screening to determine their practical value and utility.

## Introduction

Infectious diseases affecting the central nervous system (CNS) has developed a significant challenge to human health, often resulting in severe neurological complications and long-term cognitive impairments^1^. Among these diseases, herpes simplex virus-induced encephalitis (HSVE) is one of the most life thretening problem due to its capacity to induce substantial brain inflammation and damage. HSVE is primarily caused by herpes simplex virus type 1 (HSV-1) and herpes simplex virus type 2 (HSV-2)^2,3^. The most common cause of sporadic viral encephalitis is herpes simplex virus encephalitis (HSE) Despite of targeted antiviral therapy, it is still difficult to fight against HSV-1. There is evidence suggesting that chronic neuroinflammation plays a role in the development of HSV-1, even though the innate immune system is crucial for controlling HSV-1 within the brain^4^. Herpetic stromal keratitis (HSK), an immunoinflammatory condition resulting from herpes simplex virus (HSV) eye infection, can cause visual impairment. T cells are responsible for regulating this condition, and they can be reduced in size through the use of anti-inflammatory medications and treatments that modify the ratio of affected cells^5^. HSV-1 and HSV-2 infections continue to be a significant global health concern despite the availability of antiviral treatments. The increasing prevalence of drug resistance in immunocompromised patients raises serious concerns and emphasizes the urgent need for the rapid development of new, effective treatment options^6^.

Over 60% of people in the United States and 90% of people worldwide are infected with the neurotropic HSV-1. HSV-1, the leading infectious cause of encephalitis and corneal blindness, causes various biochemical and physical symptoms including cold sores and encephalitis^7,8^. While HSV-1 infection is widespread, there is currently limited vaccination or treatment available for it. Nucleoside analogs, which inhibit viral genome replication, constitute more than 75% of HSV-1 antiviral medications. Antivirals are effective in shortening the duration and frequency of outbreaks but only work against active infections; they have no impact on latent infections, which can be reactivated. The main limitations of current therapeutic approaches include the requirement for an active infection, high dosages, and the emergence of antiviral resistance. The emergence of antiviral resistance, a challenge in managing HSV-1 and other viral diseases, is significantly facilitated by targeting a shared mechanism of action. Most HSV-1 antivirals exhibit limited absorption, necessitating a high dose frequency of up to three to five times daily to maintain therapeutic levels. An exception is Valacyclovir, a pro-drug of Acyclovir, which has a better absorption profile than its parent medication and allows for a reduced dose frequency. Acyclovir, a synthetic nucleoside analog, is the cornerstone of HSV-1 infection treatment. It targets viral DNA polymerase and prevents viral replication due to its affinity for viral thymidine kinase^9,10^. The blood–brain barrier (BBB), consisting of endothelium tightly connected with glial limitans lining the CNS parenchyma, prevents pathogens from entering the CNS during infection^11^. In contrast to rabies, the breakdown of the BBB is a significant pathogenic mechanism in the development of HSE^12^. The lysis infection of neurons and glial cells, along with neuroinflammatory processes, leads to brain injury^13^. The initial release of cytokines such as type I IFN, IL-6, IL-1β, IFN-γ, and TNF results in BBB rupture when neurons and glia recognize HSV-1 through the action of toll-like receptors (TLRs)^14^.  The HSV-1 infection triggers an increased inflammatory response by attracting immune cells to the brain parenchyma, resulting in more neuronal damage. In the mouse brainstem, perivascular infiltrates near HSV-infected cells contain CD4 + or CD8 + T cells and macrophages^15^. HSV-related diseases have proven challenging to prevent and treat over the years, necessitating the development of new vaccines, or drugs.  The high infection rate of HSV makes it incurable and difficult to prevent, resulting in millions of cases worldwide^16^. The research community and scientists face a challenging task in searching for potential drug candidates. However, new drug development takes five to ten years, outpacing a single molecule, and costs an estimated $800 million to bring to market^17^. Thus, more innovative strategies are needed to find effective antiviral drugs and lower the probability of clinical trial failure. To decrease this cost burden, resource, personnel, time, and reagent waste, pharmaceutical companies have focused on computer-aided drug design (CADD) in recent years^18–20^. Using computer-aided methods makes it possible to screen molecules virtually for various pharmacological activities, saving time and money compared to more conventional methodological techniques. Therefore, this novel technology has been used in this study to identify an effective drug targeting  HSVE through Computational Drug Design Approach.

The main objective of this study to combat HSV-1 by targeting the capsid protein, the protective surface that encapsulates the virus's genetic material^21,22^. Through meticulous molecular design, our modifications are strategically crafted to infiltrate the intricate  structure of the capsid protein. This approach offers enhanced selectivity and efficacy, promising hope in the ongoing HSV-1 battle and potentially paving the way for targeted antiviral therapeutics. The capsid protein of HSV-1 is crucial in encapsulating the viral genome, protecting it during transmission and entry into host cells, making it a key target for virus replication and spread. Genetic engineering allows for modifications to prevent key interactions, improving treatment selectivity and efficacy while minimizing protein structure and function disruption. This comprehensive approach underscores the significance of targeting the capsid protein and elucidates the therapeutic strategy's promise.

## Material and methods

### Geometry optimization and ligand preparation

Density Functional Theory (DFT) was utilized in DMol3's Material Studio 08 to optimize molecules. The B3LYP functional and DND basis with semi-core pseudo-potentials and DNP + basis set were utilized. These adjustments were made with precision in mind, especially when managing electronegative atoms such as oxygen. After geometric optimization, vibrational frequencies from DMol3 were used to identify important molecular frontier orbitals (HOMO and LUMO)^23,24^. These orbitals were represented by diagrams, which are essential for comprehending chemical reactivity and electron dispersion. The study sought to obtain profound understanding of molecular behavior by computational tools, supporting additional analysis and interpretation^25,26^. Our study focused on molecular frontier orbitals after geometric optimization, identifying the HOMO–LUMO. Using vibrational frequencies taken from the DMol3 code in Material Studio 08, we carefully developed charts that displayed these important orbitals and their corresponding sizes. This detailed investigation not only revealed the complex electron inside the molecule, but it also give important new information on its electrical structure and reactivity.

### Lipinski rule and pharmacokinetics

The SwissADME online website was used to get the pharmacokinetics, drug-likeness score, and characteristics (http://www.swissadme.ch/index.php)^27^. This online repository contains all the information needed for developing new medicines. To describe the drug-likeness metrics of ligands, the following data were measured: topological polar surface area (TPSA) 2, molecular weight, hydrogen bond donors (HBD), hydrogen bond acceptors (HBA), bond rotation numbers (NRB), and lipophilicity.

### ADMET profile prediction

Absorption, distribution, metabolism, excretion, and toxicity are all referred to as ADMET. This parameter has been regarded as the most important and potentially useful for drug discovery and development. The Swiss ADME and pkCSM online tools at “http://biosig.unimelb.edu.au/pkcsm/predictionsingle/adme1643650057.59” have provided the ADMET measurements^28^. Although this web tool reports on between 60 and 70 characteristics. The BBB, water solubility, total clearance, maximum tolerance rate, hepatotoxicity, oral rat acute toxicity (LD50), oral rat chronic toxicity, skin sensitization, etc., are just a few of the potential factors that we have concentrated on.

### Target structure preparation and molecular docking analysis

The capsid protein of HSV-1 (PDB ID: 1NO7) was downloaded from data bank (PDB) (http://www.rcsb.org)^29^. Following that, the water molecules found in the protein, heteroatoms, and other materials were effectively removed, and the prepared protein using the discovery studio application. Finally, this prepared protein was saved in PDB format. Next, the PyRx application was utilized to conduct molecular docking analysis using the AutoDock vina functionalities, allowing for the determination of the binding affinity to each ligand. When conducting molecular docking experiments, the protein was entered as a macromolecule, while the ligand was entered as a ligand. After reloading the ligand, the optimization process focused on maximizing energy and grid surface area. This involved adjusting the center and dimensions of the grid^30,31^. Once the docking experiment was done, the dock molecules were picked up and uploaded into the Pymol software version PyMolV2.3 (https://pymol.org/2/) and generated a complex for molecular dynamic simulation^32,33^. Then, the ligand–protein docking complex’s active side and non-covalent contacts were identified using Discovery Studio visualization and ChimeraX at the endpoint.

### Molecular dynamic simulation

The molecular dynamics simulations were conducted using the Assisted Model Building with Energy Refinement (AMBER) 14 force field and Yet Another Scientific Artificial Reality Application (YASARA) dynamics program^34^. The hydrogen bond network was cleaned up and optimized along with the docked complexes. The General AMBER Force Field (GAFF) was used to create the ligands' topology files, and AM1BCC charges were assigned. The TIP3P solvation model with periodic boundary conditions was used to generate a cubic simulation cell^35^. The simulation parameters were set to 0.9% NaCl, 310 K, and pH 7.4 to produce a neutral system^36^. With an 8.0 cutoff radius, the particle mesh Ewald (PME) approach was used to calculate the long-range electrostatic interactions. The simulation was run with a 2.0 fs time step. Using the steepest gradient algorithm and simulated annealing techniques (5000 cycles), the primary energy minimization was carried out. The simulated trajectories were used to analyze a number of characteristics, including RMSD, Rg, SASA, hydrogen bonds, RMSF, and MMPBSA binding free energy. Every 100 ps, during the simulation's 100 ns runtime, a snapshot of the trajectory was taken^37^.

## Result analysis

### Structural activity relationship (SAR) studies

**Figure 1:**
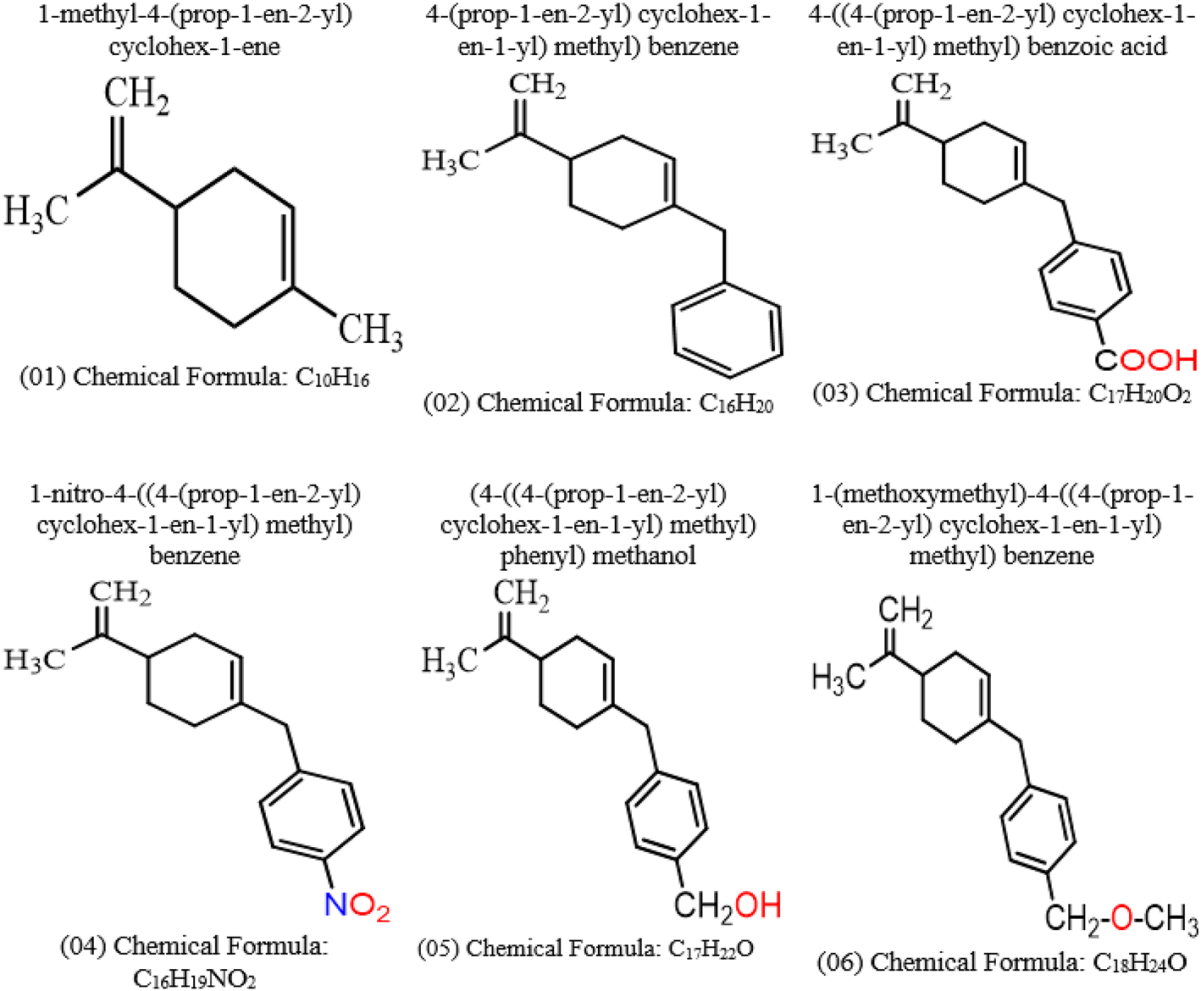
Chemical structure of limonene and its derivatives.

The structure–activity relationship (SAR) enables desired effects during the development of the new drug, such as identifying new drug candidates and enhancing their desirable functionalities. This method is gaining popularity day by day to design specific drug molecules against certain diseases. Limonene has already been reported as broad-spectrum antimicrobial properties. So, the natural limonene is modified by adding different functional groups such as benzene, COOH, NO_2_, CH_2_–OH, and CH_2_–O–CH_3_ to get better efficiency. After modification of the structure of limonene, it is observed that the efficiencies and pharmacokinetics are improved against HSV-1 targeted protein. Besides, the mentioned functional group strongly impacts aqueous solubility, ADMET, and binding affinities. So, it is clearly understood that the structural activity relationship plays a vital in finding new bioactive molecules. The main objective is to determine how different functional groups impact the drug properties of limonene. The modified structures are given in Fig. 1.

### Pharmacokinetics and drug likeness

The evaluation of the possibility of a specific biochemical to be utilized as an oral medication relating to bioavailability and physicochemical properties is known as pharmacological drug-likeness, determined by the SwissADME web tool^25,27^. This is a crucial investigation since it is believed that nine out of ten targeted drugs do not reach the final stages due to their adverse effects, which results in vast amounts of wasted expenses, time, and human resources^38^. This issue arises because there is a lack of proper identification of the suitable physiochemical features of medication. The Lipinski rule is the fundamental and novel strategy to reduce the chance of failing, also called drug likeness^39^. This investigation mentioned that all the ligands have a potential bioavailability score (0.55–0.85), whereas only ligand 04 has a maximum bioavailability score of 0.85 due to carboxylic group attachment. Besides, the number of rotatable bonds**,** hydrogen bond acceptor, and hydrogen bond donor ranges are 01–04 for all ligands where the molecular weight is about 136.23 Dalton–257.33 Dalton. Finally, all the drugs accepted druglikeness parameters such as Lipinski rule for all ligands. Therefore, it is reasonable to conclude that the ligands might be used without risk. The conclusive findings of the pharmacokinetics and drug-likeness analyses are shown in Table 1.

**Table 1:** Data of Lipinski rule, pharmacokinetics, and drug-likeness.

Ligand no.	Molecular weight	Number of rotatable bonds	Hydrogen bond acceptor	Hydrogen bond donor	Topological polar surface area Å^2^	Druglikeness	
						Lipinski	violation
01	136.23	01	00	00	0.00	Yes	0
02	212.33	03	00	00	0.00	Yes	0
03	256.34	04	02	01	37.30	Yes	0
04	257.33	04	02	00	45.82	Yes	0
05	242.36	04	01	01	20.23	Yes	0
06	256.38	05	01	00	9.23	Yes	0

### Molecular docking against capsid protein of Herpes Simplex Virus-1

The molecular docking has been conducted to determine the binding affinity when drug protein make complex each other. The generated pharmacological data of binding affinity was determined using molecular docking computations. In these simulations, bioactive components of the ligands interact with a peptide from capsid protein of HSV-1. The protein–ligand interaction is essential in development, especially in in silico and virtually screening drug development^40,41^.

In our investigation, the initial docking score was − 5.6 kcal/mol against capsid protein of HSV-1. But, with the addition of a functional group or side chain, the docking score has gradually increased and crossed the FDA-approved standard Acyclovir score.

The predicted score has displayed in Table 2 for better understanding. In details description, the molecular docking score ranges for capsid protein of HSV-1, and they all are showing the better binding impact than the standard Acyclovir. So, they could be regarded as better compounds than the standards. It is noted that when we have modified the primary structures of limonene, the binding affinity was obtained better effectiveness in comparison with the primary structures of limonene, and it is said that the functional groups play significant roles to be effectiveness, and potentiality of binding affinity. Hence, overall, the finding reported that ligands 02 and 03 are the most active molecules, and due to the addition of Benzene ring and –COOH functional group.

**Table 2:** Binding affinity against gram-positive and gram-negative bacteria.

Molecules no.	Capsid protein of Herpes Simplex Virus-1 (PDB ID 1NO7)
	Binding affinity (kcal/mol)
01	− 5.6
02	− 7.4
03	− 7.1
04	− 6.7
05	− 6.9
06	− 6.5
Standard acyclovir	− 6.5

### Protein–ligand interaction, molecular docking poses, and active site analysis

**Figure 2:**
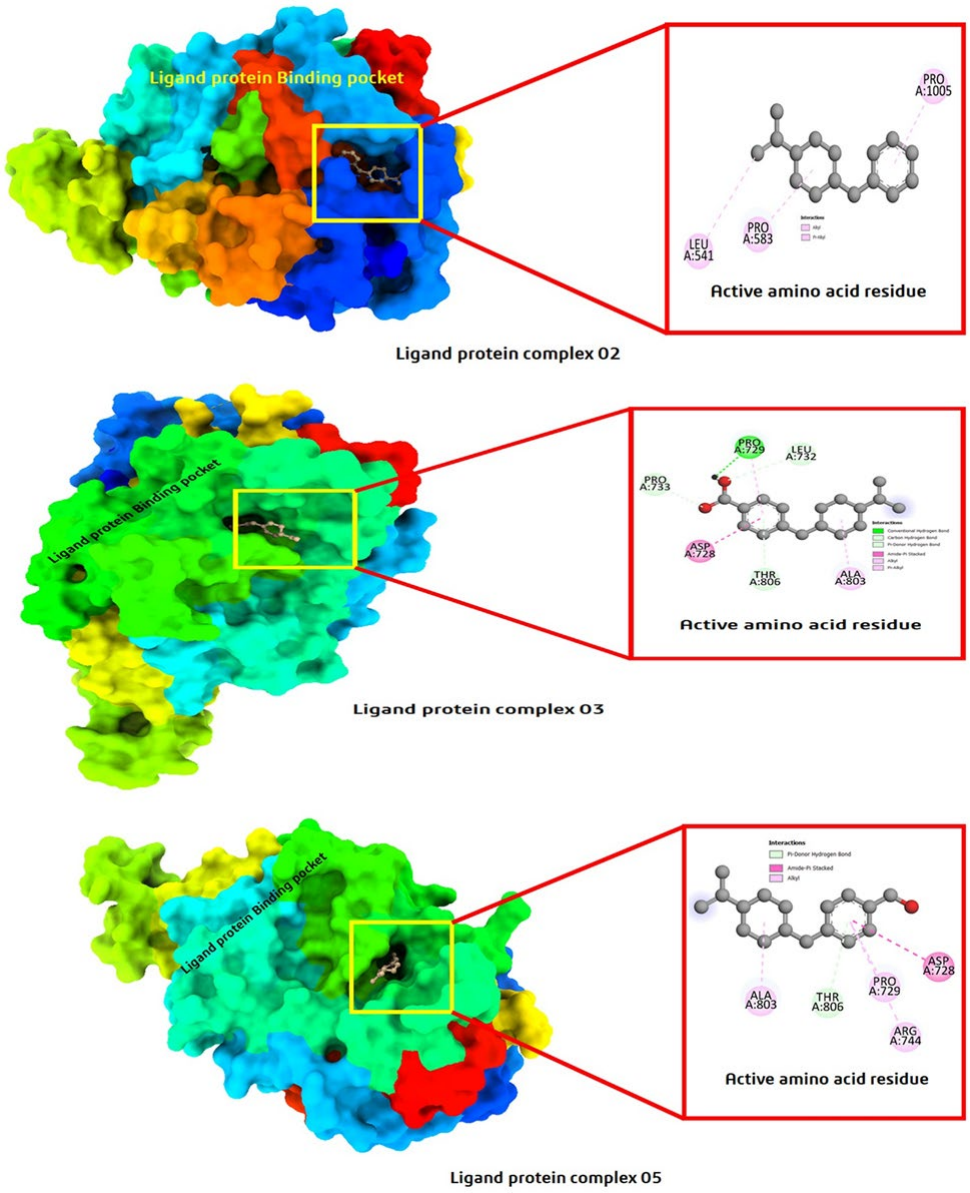
Protein–ligand interaction diagram and active site analysis. The Pink colour represent Pi-alkyl bonds, and the deep green colour hydrongen bonds.

The most significant point to note when developing a novel drug is how a weak or covalent bonds between a ligand and a protein is configured. This investigation approximates the binding affinity or energy of various substances with the proteins of the pathogens. Measurements of the bond distance, active side, and protein ligand interaction aided researchers better understand how the chemical and the protein interact with each other. According to the graphical representation, the protein–ligand complex was actively generated by the ligand and protein, which appeared to form pockets and display various active amino acid residues. With the help of Biovia Discovery Studio version 2021^42^, this entire interaction was formed based on the highest docking score, and given in Fig. 2. The docking pocket represents how a drug binds with ligands and in which position of protein they are engaged.

### Frontier molecular orbitals and quantum calculation

In broad terms, chemical descriptors are essential to any organic or physiologically active drug-like compound. The Highest Occupied Molecular Orbital (HOMO) orbital is the one that might function as an electron donor due to being the highest energy orbital that encompasses electrons. On the other hand, the Lowest Unoccupied Molecular Orbital (LUMO) orbital is the one that might function as an electrophile because it is the lowest energy orbital that has space to receive electron^42,43^. The magnitudes of εLUMO, εHOMO, and the energy gap differentiation (Δε), hardness (η), and softness (S) of the six molecules 01–06 are displayed in Table 3. These data estimates were computed using the B3LYP functional.

**Table 3:** Data of chemical descriptors.

Drug no.	A = − LUMO	I = − HOMO	Energy = I–A	Hardness	Softness
01	− 0.704	− 8.397	7.693	3.846	0.2600
02	− 0.363	− 7.537	7.174	3.587	0.2788
03	− 0.206	− 7.608	7.402	3.701	0.2702
04	− 0.090	− 7.811	7.721	3.8605	0.2590
05	− 0.270	− 7.402	7.132	3.566	0.2804
06	− 0.076	− 7.688	7.612	3.806	0.2627

Comparing the molecular frontier orbital properties of six drugs (01 to 06) against accepted standard ranges provides insights into their reactivity and pharmacological potential. The negative values of both LUMO and HOMO across all drugs indicate their ability to accept and donate electrons, respectively, for drug interaction. The relatively small energy gaps suggest moderate to high reactivity. Furthermore, hardness values ranging from 3.566 to 3.8605 imply moderate stability, while softness values between 0.2590 and 0.2804 indicate moderate reactivity. Overall, these findings contribute to understanding the pharmacological characteristics and reactivity profiles of the drugs, facilitating their development and optimization.

We find consistent trends that reflect the electrical characteristics of the six medications when we compare their HOMO and LUMO values. The range of values for the HOMO is − 7.402 to − 8.397, and the range for the LUMO is − 0.090 to − 0.704. These negative values imply that all medications have electron-donating (HOMO) and electron-accepting (LUMO) properties, which are advantageous for molecular interactions. Furthermore, there is moderate to high reactivity indicated by the relatively modest energy gaps between HOMO and LUMO, which range from 7.132 to 7.721 (Table 3). All medications have constant negative values for HOMO and LUMO, indicating their ability to donate and take electrons, which is essential for pharmacological interactions and reactivity^44,45^.

Therefore, these compounds may have potential as drug candidates. Additionally, the softness values range from 0.2590 to 0.2804; generally, drugs with lower softness values decompose more quickly.

On the other hand, one of the essential characteristics of a material is its hardness, which reflects its resilience. A higher hardness value indicates greater strength and stability of the compound. The information in Table 3 reveals that hardness is often correlated to the energy gap. Ligand 05 shows the lowest hardness value, suggesting that this material has slightly inferior stability and softness, which means it may decompose more rapidly than others. Supplementary Fig. S1 has shown the Frontier Molecular Orbitals (HOMO and LUMO) diagram.

### Molecular of electrostatic potential (MEP) charge distribution map

Molecular Electrostatic Potential (MEP) is a valuable tool for integrating various physicochemical features of drug molecules, such as dipole moment, electronegativity, and partial charges. It provides insights into the electrophilic and nucleophilic regions of compounds. By analyzing the distribution of potential energy formed by the molecule's electrons and nuclei, MEP serves as a static dispersion of potential and aids in defining and predicting the functional behavior of a compound. Electrostatic potentials have played a significant role in investigating diverse phenomena in biological, physical, and related fields.

Figure 6 illustrates the dynamically calculated MEP values for compounds (01–06) of limonene derivatives, which were predicted using the DFT method. In this representation, the red color indicates the lowest electrostatic potential energy intensity, representing the most significant negative area and the location most susceptible to electrophilic attacks. Conversely, the blue zone in the MEP signifies the maximum value of the potential electrical charge, indicating both the maximal positive region and a place favorable for nucleophilic substitution. The white color represents no potential or zero potential, as demonstrated in Supplementary Fig. S2.

Overall, the red and blue zones on all the molecules are identical, suggesting that these chemicals are almost equally prone to electrophilic reactions and nucleophilic attacks. However, a crucial finding indicates that their red regions are considerably more significant than their blue regions, highlighting their higher propensity for electrophilic reactions".

### ADMET data investigation

When it comes to drug development, using techniques such as in silico pharmacokinetics and ADMET analysis is  essential. These approaches facilitate determine the chemical and physical characteristics of compounds, ensuring the process more efficient and cost-effective^46,47^. Using computational tools like SwissADME and pkCSM, we executed ADMET analysis on limonene derivatives (Ligand 01–06)^48^. This investigation is essential to evaluate pharmacokinetics properties such as absorption, distribution, metabolism, excretion, and toxicity. These factors have significance to maintaining the efficacy and effectiveness of potential oral medications. The investigation addressed a wide range of factors related to the behavior of these substances. This included their solubility in water, how easily they can pass through Caco-2 cells, their absorption rates in the gastrointestinal tract, and any potential interactions with metabolic enzymes and transporters.

When it comes to absorption, understanding the water solubility (Log S) values may provide us useful information about how well compounds may disintegrate in water. This is essential because it affects how well they are absorbed in the gastrointestinal tract^49^. Based on the results of this study, it can be observed that Ligands 02 and 06 have Log S values indicating moderately solubility. On the other hand, the other compounds show better soluble characteristics which implies a promising potential for absorption. The solubility is ranges are considered by the Log S scale: insoluble < − 10 poorly < − 6, moderately < − 4 soluble < − 2 very < 0 < highly^50–52^.

It is important to note that positive Caco-2 permeability values indicate efficient absorption across intestinal epithelial cells. This further supports the compounds' suitability for oral administration through assuring their beneficial passage through the intestinal barrier^53,54^.

In addition, the results indicate positive findings regarding distribution, metabolism, and excretion (Table 4). It is worth mentioning that the compounds exhibit remarkable rates of GI absorption, with Ligands 03, 04, and 05 achieving full absorption (100%). Furthermore, the molecules' potential to cross the blood–brain barrier highlights their possible therapeutic benefits, with the exception of Ligand 02. When it comes to metabolic considerations, there are interesting interactions with CYP450 enzymes. It's fascinating to observe how different compounds can exhibit inhibition of CYP450 1A2 while not affecting 2C9. This data is essential for predicting possible drug-drug interactions and providing guidance for dosage adjustments.

**Table 4:** Summary of calculation of ADME results for selected Limonene derivatives.

S/N.	Absorption			Distribution		Metabolism		Excretion	
	Water solubility Log S	Caco-2 ppermeability (10–6 cm/s)	Intestinal absorption (human) %	VDss (human) (log L/kg)	B.B.B permeability	CYP450 1A2 inhibitor	CYP450 2C9 Inhibitor	Total clearance (ml/min/kg)	Renal OCT2 substrate
01	− 3.568	1.403	95.898	0.396	Yes	No	No	0.214	No
02	− 5.546	1.560	95.125	0.92	No	Yes	No	0.187	No
03	− 2.252	1.719	100	− 1.411	Yes	No	No	0.643	No
04	− 2.683	1.312	100	− 1.602	Yes	No	No	− 0.003	Yes
05	− 2.656	1.263	100	0.039	Yes	No	No	− 17.034	No
06	− 5.502	1.527	95.883	0.906	Yes	Yes	No	0.314	No

In addition, analyzing the overall clearance rates offers valuable information about how quickly the compounds are eliminated from the body. The clearance ranges are consistently favorable, suggesting efficient elimination. It is important to mention that Ligand 04 might be excreted through the kidneys using the OCT2 substrate pathway. This indicates the importance of monitoring patients with impaired renal function. In general, conducting thorough ADMET evaluations is essential for understanding the safety and pharmacokinetic properties of limonene derivatives. These theoretical ADMET finding might be helpful to the researcher for the subsequent stages of preclinical and clinical development, with a particular emphasis on improving efficacy and safety.

### Aquatic and non-aquatic toxicity

Table 5 lists the aquatic and non-aquatic toxicities, which are essential for determining whether drugs or other substances are tolerable in the environment before and after use^25^. When directly indicated, none of the chemicals exhibit hepatotoxicity or toxicity to AMES., indicating that they will not be carcinogenic to humans or laboratory animals or induce liver toxicity. The vast majority of drugs, except for compounds 04 and 05, caused by AMES. The finding has been reported that the max. tolerated dose ranges from 0.309 mg/kg/day to 0. 770 mg/kg/day, oral rat acute toxicity (LD50) range from 1.747 mol/kg to 2.482 mol/kg. Finally, the oral rat chronic toxicity range is about 1.037 mg/kg/day to 4.689 mg/kg/day for all compounds. Therefore, it might be concluded that they do not threaten humans or the environment, and further experimental studies need to be established as safe medication.

**Table 5:** Aquatic and non-aquatic toxicity value prediction.

S/N.	AMES toxicity	Max. tolerated dose (human) mg/kg/day	Oral rat acute toxicity (LD50) (mol/kg)	Oral rat chronic toxicity (mg/kg/day)	Hepatotoxicity
01	No	0.770	1.88	2.368	No
02	No	0.526	1.747	1.343	No
03	No	0.490	2.117	2.085	No
04	Yes	0.309	2.482	4.689	No
05	Yes	0.347	2.482	2.244	No
06	No	0.690	1.837	1.037	No

### Molecular dynamic simulation result analysis

#### Root mean square deviation (RMSD), and radius of gyration (Rg) analysis

**Figure 3:**
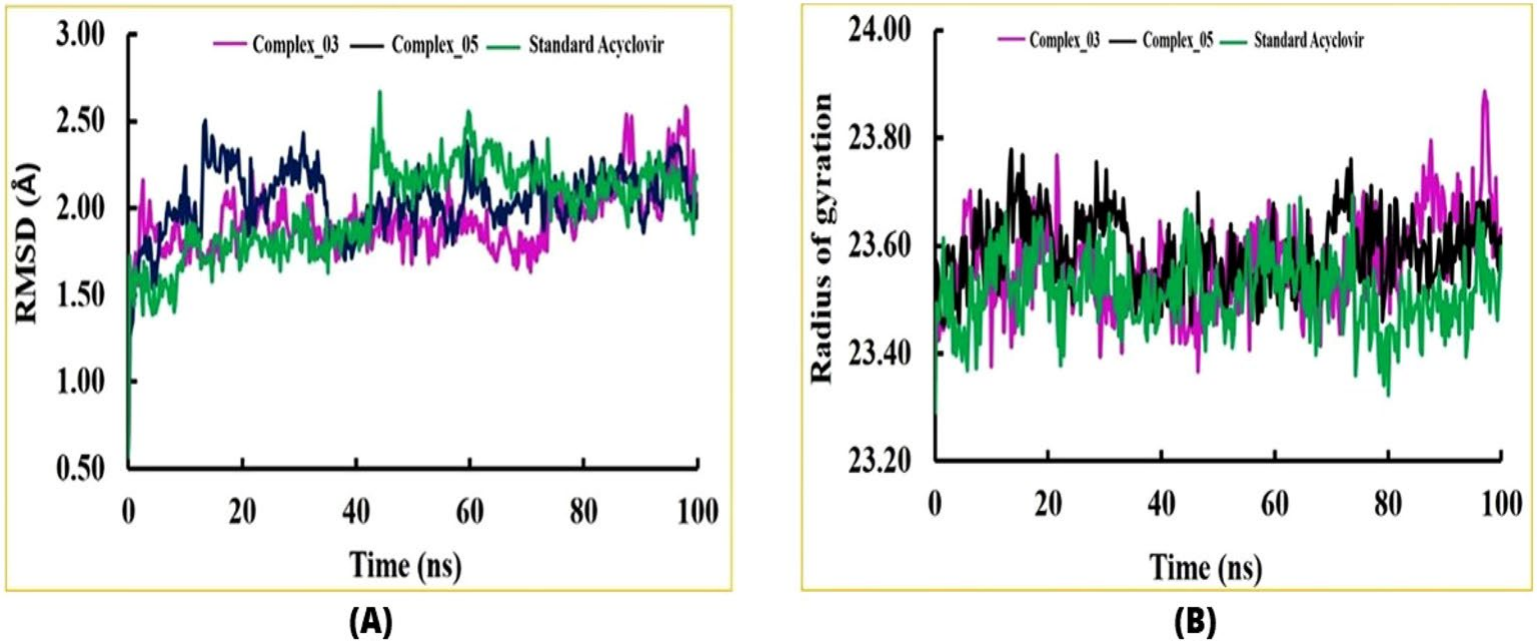
Two key metrics: (**A**) Root Mean Square Deviation (RMSD), and (**B**) Radius of Gyration (Rg). These analyses were carried out on complexes formed by limonene derivatives, specifically complex_03 and complex_05, in comparison to the standard compound Acyclovir. In our visual representations, complex_03 is denoted by a pink color, complex_05 by black, and Acyclovir by green. By investigating the RMSD and Rg values, we aimed to discern any significant differences or similarities between the behavior of the limonene derivatives complexes and the standard Acyclovir throughout the duration of the molecular dynamics.

MD simulation was conducted to ensure the stability of drug-protein complex at 100 ns^55,56^. In this work, we quantitatively evaluated structural alterations, stability, and dynamics in biomolecular systems using RMSD analysis, a critical tool in molecular dynamics simulations. In particular, we performed an RMSD study on complexes (called complex_03 and complex_05) that were generated between the HSV-1 capsid protein and two limonene derivatives. As shown in Fig. 3A, these profiles were compared to those of the regular Acyclovir. The mean RMSD values that were acquired were 1.92 Å for complex_03 and 2.04 Å for complex_05. These results were in close agreement with the conventional Acyclovir's RMSD value of 1.99 Å. We saw an early increase in RMSD values for all complexes during the first few nanoseconds of the experiment. After this first spike, though, the complexes showed a tendency to hold onto stability, while little  fluctuations were noticed here and there over the experiment. Like Fig. 3A illustrates, these variations often fell between 1.50 and 2.50 Å. Despite the small variations between complexes, they did not significantly impact overall stability or cause significant structural changes.

Secondly, Rg analysis is a versatile tool in MD simulations that provides valuable information about the structural dynamics and compactness of biomolecules. It aids in understanding conformational changes, stability, interactions, and folding processes, making it an integral part of drug discovery focused on molecular dynamics studies^9^. Initially, the radius of gyration (Rg) profiles for all three complexes exhibited an upward trend up to 20 ns (ns). Subsequently, these profiles maintained a stable configuration from 20 to 65 ns. However, after a slight downward movement between approximately 75 ns and 83 ns, an increase in Rg values was observed. Of particular interest is the fact that all of the complexes displayed a similar pattern in their Rg profiles when compared to the standard. The observed pattern in the Rg profiles suggests a common behavior across all complexes and the standard. The initial upward movement in Rg indicates a phase of structural expansion or rearrangement within the first 20 ns. The subsequent stable profile from 20 to 65 ns implies that the complexes reached a relatively equilibrium-like state, characterized by minimal changes in their overall size and conformation. The subsequent increase in Rg values after the slight downward movement between 75 and 83 ns could indicate a phase of structural changes or unfolding. The consistent pattern among the complexes and the standard suggests that there might be shared underlying factors influencing their dynamics (Fig. 3B).

#### Solvent accessible surface area (SASA) analysis

The SASA analysis serves to quantify the fraction of a molecule's external surface that remains exposed and open for attachment with surrounding solvent molecules. This investigation is particularly significant in understanding protein stability and folding dynamics^10^.

**Figure 4:**
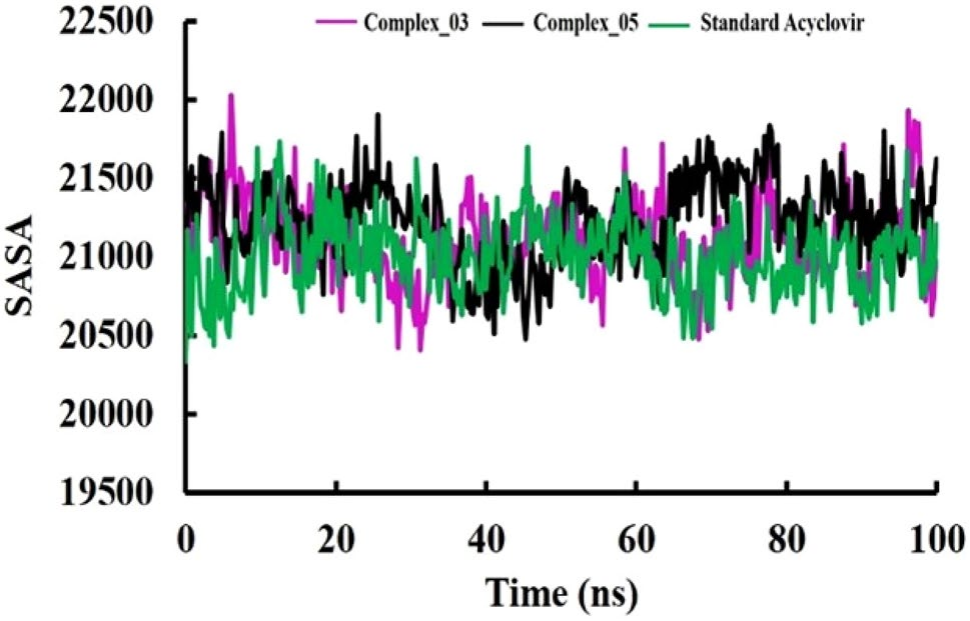
Solvent accessible surface area (SASA).

During our study, the SASA profiles of both complex_03 and complex_05 exhibited a consistent level of stability, marked by minor fluctuations. Interestingly, this stability closely resembled the SASA profile of the standard Acyclovir (Fig. 4). This observation indicates that the surface accessibility of solvent molecules to complexes complex_03 and complex_05 remained relatively unchanged, with only slight variations throughout the simulation. Remarkably, this pattern of stability mirrored the behaviour seen in the SASA profile of the standard Acyclovir. In essence, the SASA profiles suggest that the external surfaces of all three entities complex_03, complex_05, and Acyclovir interacted with the surrounding solvent molecules in a comparable manner. This consistent trend may imply a shared mode of surface interaction, possibly linked to similar structural characteristics or dynamic behaviours among the molecules. This aims to assess the level of surface accessibility to solvent molecules for each compound. We compared the SASA values of limonene derivatives complexes and Acyclovir to identify any significant differences in their behavior during the simulated period.

#### Hydrogen bonds analysis

**Figure 5:**
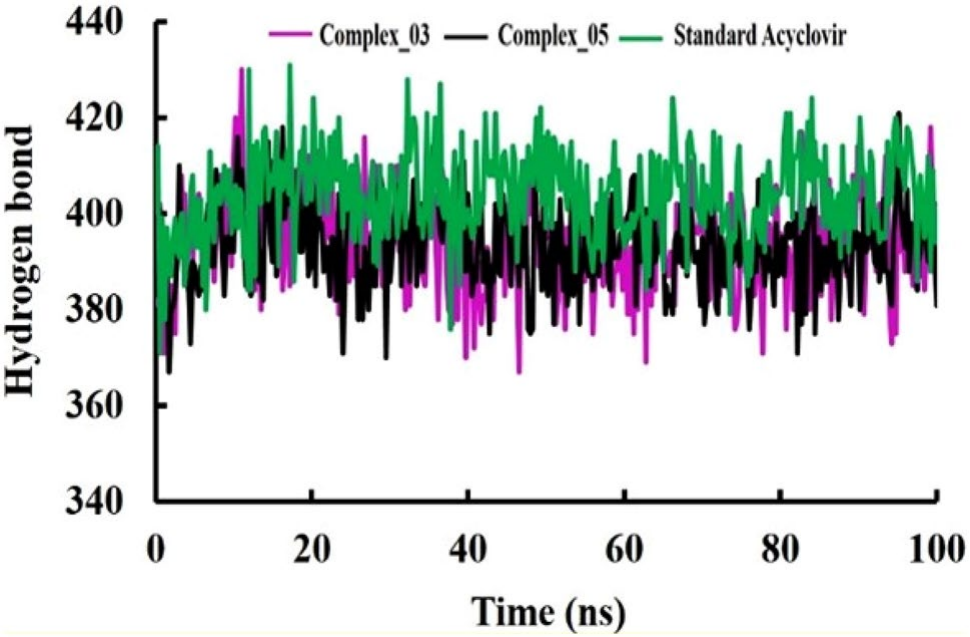
The hydrogen bonds within complexes formed by limonene derivatives complex_03 and complex_05, comparing them to the standard Acyclovir. This analysis aimed to elucidate the hydrogen bonding patterns and dynamics within each complex.

Hydrogen bond analysis is an essential component of molecular dynamics (MD) simulation studies due to the significant role that hydrogen bonds play in shaping the structure, dynamics, and interactions of biomolecules. Based on our discoveries, we observed that the hydrogen bond patterns for all three complexes initially showed an upward trend up to 15 ns. This finding implies that during the initial phase of the simulation, hydrogen bonds were actively forming, potentially indicating structural adjustments or interactions among the components. Subsequently, complex_03 and complex_05 maintained stable hydrogen bond profiles that were notably similar throughout the entire 100 ns simulation period. This stability closely resembled the hydrogen bond profile of the standard Acyclovir. However, it's worth noting that the standard Acyclovir exhibited slightly higher hydrogen bond values compared to the other two complexes throughout the simulation (Fig. 5). The subsequent stability in hydrogen bond profiles for complex_03 and complex_05 suggests that these complexes stable into configurations where their hydrogen bonding patterns remained relatively constant over time. Furthermore, the similarity in hydrogen bond profiles between complex_03, complex_05, and Acyclovir underscores a potential commonality in their structural behaviors or interactions.

#### Root-mean-square fluctuation (RMSF) analysis

**Figure 6:**
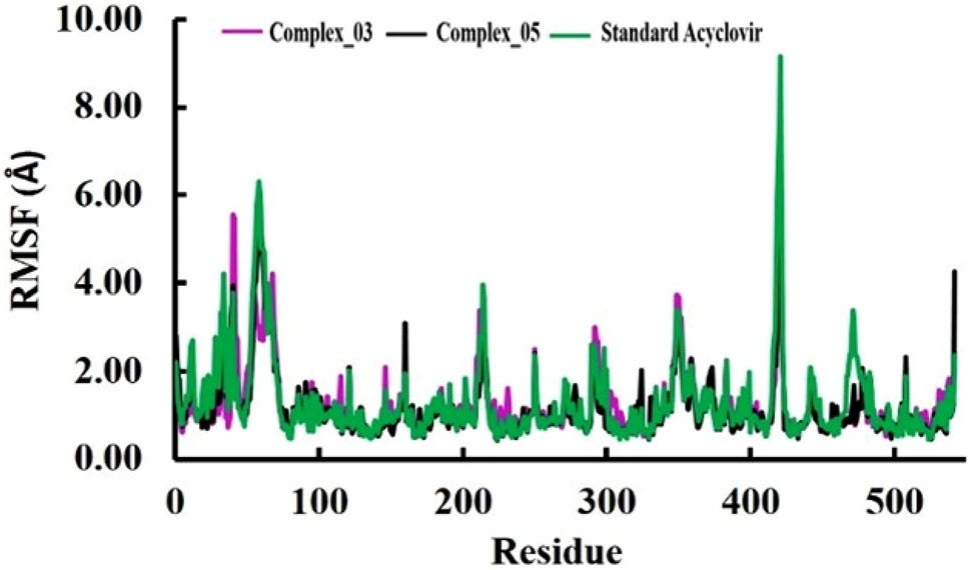
The analysis of Root-mean-square fluctuation (RMSF). The RMSF analysis enabled us to investigate the flexibility and dynamics of the protein–ligand complexes. We analyzed the differences in binding stability and dynamics between the limonene derivatives complexes and Acyclovir by comparing their RMSF profiles.

In this study we used RMSF to investigate the flexibility and dynamic behavior of each of the atoms or residues within a biomolecular system, it is also utilized to identify specific residues that contribute to these fluctuations. The two-limonene derivatives complex_03 and complex_05 showed an excellent level of similar pattern RMSF profile with standard Acyclovir. However, some residue fluctuated more than 4 Å such as Arg34, Leu41, Leu58, Asp62, Pro63, Gly215, Pro349, Ala418, Asn419, Thr420, Ala421, and His542 (Fig. 6). The combined similarity in RMSF profiles along with the notable flexible behavior of specific residues provides valuable insights into the dynamic behavior of the complexes. Despite the overall similarity in RMSF profiles, certain residues within these complexes demonstrated a higher degree of flexibility or mobility during the simulation. The residues mentioned, such as Arg34, Leu41, and others, could potentially be part of flexible loops, active site regions, or points of interaction within the complexes. The increased flexibility in these residues might have functional implications, such as facilitating binding events, accommodating conformational changes, or participating in molecular recognition processes.

#### MMPBSA binding energy

**Figure 7:**
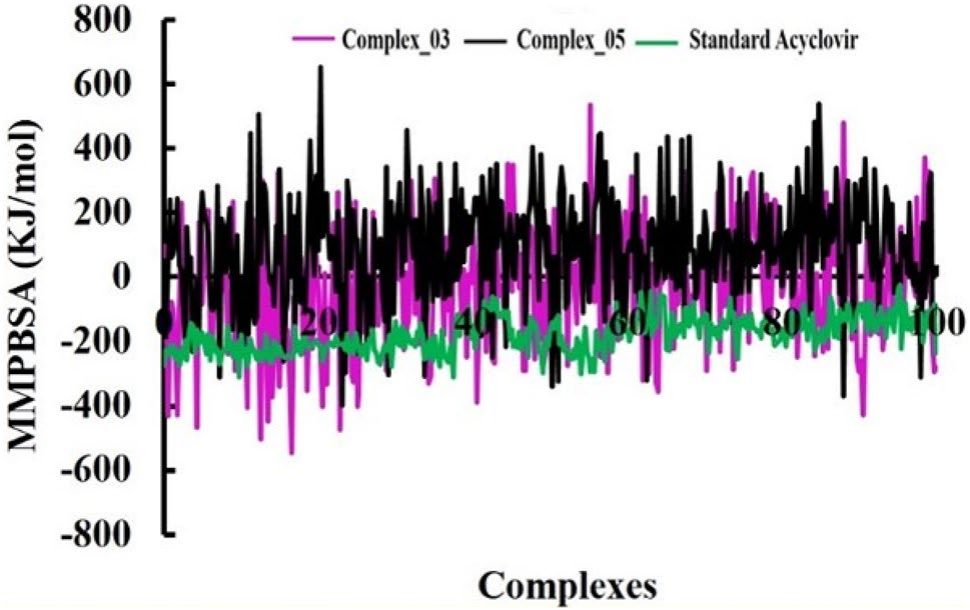
The analysis of MMPBSA analysis binding energy at 100 ns molecular dynamics simulations periods.

The MMPBSA binding energy is a quantitative measure of the net energy change that occurs when a protein–ligand complex is formed compared to the individual unbound components. In our investigation, we utilized Molecular Mechanics/Poisson-Boltzmann Surface Area (MMPBSA) calculations to  investigate the binding free energy associated with protein–ligand interactions. These MMPBSA binding free energies were computed for all complexes over a 100 ns simulation period, as depicted in Fig. 7. Our study revealed that the average MMPBSA binding energies for the three complexes complex_03, complex_05, and standard Acyclovir were calculated as − 22.94, 91.35, and − 178.81 kJ/mol, respectively. Throughout the study of the 100 ns simulation period, the MMPBSA binding energy profiles for the three complexes exhibited distinct patterns of stability. Notably, despite this variation in profiles, the complexes' binding energies remained relatively constant over time. These findings imply that complex_05 exhibited a relatively weaker binding interaction compared to complex_03 and standard Acyclovir. Acyclovir, the standard reference, exhibited the strongest binding interaction, indicated by a significant negative MMPBSA binding energy.

## Discussion

The four layers that make up the HSV-1 particle include an inner core that families the viral DNA, a protein shell known as the capsid, a multiprotein layer known as the tegument, and an envelope made of viral glycoproteins that are generated from cellular membranes. The most widely recognized theory explaining HSV-1 postulates that capsids are formed in the nucleus, integrate viral DNA, fuse with the outer nuclear membrane to lose the first envelope, and then emerge into the cytoplasm nude^57–59^. The Herpesviridae family is associated with various diseases such as infectious mononucleosis, nasopharyngeal cancer, Kaposi's sarcoma, and herpes lesions of the genitalia and lips^21^. All three herpesvirus subfamilies—α-, β-, and γ-herpesviruses—have nine known human herpesviruses. These viruses are categorized based on biological characteristics, such as variations in tissue tropism. The α-herpesvirus subfamily of viruses includes varicella-zoster virus (which causes chickenpox and shingles), herpes simplex virus type 1 (HSV-1, causes cold sores), and type 2 (HSV-2, causes genital herpes). These viruses can cause lifelong latent infections in the peripheral nervous systems of their hosts.

The study of HSV-1 structure-activity correlations is an essential for developing advanced antiviral drugs that specifically target the virus. Through comprehension of the ways in which specific structural characteristics influence the effectiveness and specificity of existing antiviral medications, scientists can develop novel molecules that possess enhanced potency and reduced off-target effects. Multi-targeted medicines can be developed as a result of investigating the connections between molecular structures and antiviral activity, which may uncover synergy between various therapeutic targets or pathways involved in the virus's replication cycle. By utilizing structure–activity insights to optimize pharmacokinetic features, these molecules can further boost their potential for therapeutic use, and possible to optimize pharmacokinetic features, the molecules in question can further improve their potential for therapeutic use in vivo^60,61^. These discoveries also provide new molecular targets and pathways for preventing HSV-1 proliferation, opening the door for new antiviral treatments. All things considered, utilizing the structure–activity knowledge gained from HSV-1 research should transform the development and refinement of next-generation antiviral drugs, providing a more effective means of combating HSV-1 infections.

One well-known cyclic monoterpene is limonene. This hydrocarbon is an olefin (C_10_H_16_), and it exists in two optical forms. (++)-One of the most significant and widely used terpenes in the flavor and fragrance industry is limonene. Limonene (both optical forms) is present in over 300 plant essential oils derived from a wide range of species, such as fir, orange, lemon, and mint^62^. One of the most common smells used in cosmetic formulations, limonene is a monoterpene that is mostly found in cleaning and food products. Limonene has the therapeutic effects such as anti-inflammatory, antioxidant, antinociceptive, anticancer, antidiabetic, antiviral, and gastroprotective properties^63^. Essential oils are complex combinations of constituents from multiple functional group groups. The system can differentiate between monoterpenes, phenylpropanes, and other constituents based on their structural composition. The study tested the antiviral properties of monoterpene compounds, limonene and β-pinene, in essential oils against HSV-1 in vitro. Both limonene and beta-pinenene completely eliminated viral infectivity. Monoterpenes have been identified as having an antiviral action mechanism, but they only show moderate activity when administered to host cells before or after HSV entry. Both monoterpenes demonstrated strong anti-HSV-1 efficacy upon direct attachment with free virus particles. The viral infection was reduced inactive by the dose-dependent interactions between the two studied medications and HSV-1^64^. The main objective of our study was to identify how structural modification, and different function group can affect the binding affinity, and pharmacological effectiveness against capsid protein of HSV-1. Our study found that addition of different functional group can improve the binding affinity compare to the primary compounds. Now, according to the literature finding, and our in silico study both can be compared, and concluded that Limonene can actively inhibit the capsid protein of HSV-1, and fulfil the main objective the investigation.

## Conclusion

This research has amplified and integrated limonene derivatives as an innovative antiviral drugs using computer-aided methodologies and performed different computational approaches against capsid protein of HSV-1*.* After a comprehensive investigation, it is reported that the maximum binding affinity score for capsid protein of HSV-1 has been displayed − 7.4 kcal/mol and − 7.1 kcal/mol. Besides, the drug-likeness of all compounds is entirely accepted. Therefore, the drug development and implementation process may be guided by anticipating the in silico modeling of ADMET features for safe medicine.

Additionally, it obtained an improved ADMET profile for all drugs with low toxicity and greater to moderate solubility; most of the medication can cross the BBB and may be metabolized by CYP450 1A2 inhibitor. The chemical descriptors HOMO–LUMO reported that the energy gap for ligands 01–06 is around 7.174–7.693, while the softness value is between 0.2590 and 0.2804. Finally, molecular dynamic modeling has demonstrated that they are stable drugs in many conditions and ought to demonstrate improved stability once they enter the biological system. Therefore, it may be inferred that researcher searching for antiviral medications against for capsid protein of HSV-1 are capable of developing innovative drug.

### Supplementary Information

Supplementary Figures.

## Data Availability

All data are available in this manuscript.
