# Author Correction: Ligand-based drug design against Herpes Simplex Virus-1 capsid protein by modification of limonene through in silico approaches

**DOI:** 10.1038/s41598-024-61227-8

**Published:** 2024-05-08

**Authors:** Md. Rezaul Islam, Md. Shafiqul Islam Sovon, Ummy Amena, Miadur Rahman, Md. Eram Hosen, Ajoy Kumer, Mohammed Bourhia, Yousef A. Bin Jardan, Samir Ibenmoussa, Gezahign Fentahun Wondmie

**Affiliations:** 1 Department of Pharmacy, Faculty of Allied Health Sciences, Daffodil International University, Dhaka, Bangladesh 1207; 2 Department of Pharmacy, Faculty of Pharmacy, University of Dhaka, Dhaka, 1000 Bangladesh; 3 Department of Pharmacy, Faculty of Life & Earth Sciences, Jagannath University, Dhaka, Bangladesh; 4 Department of Pharmaceutical Sciences, North South University, Dhaka, 1219 Bangladesh; 5 Department of Genetic Engineering and Biotechnology, University of Rajshahi, Rajshahi, 6205 Bangladesh; 6 Department of Chemistry, College of Arts and Sciences, International University of Business Agriculture and Technology (IUBAT), Dhaka, 1216 Bangladesh; 7 Center for Global Health Research, Saveetha Institute of Medical and Technical Sciences in Saveetha Medical College and Hospital, Chennai, India; 8 Laboratory of Biotechnology and Natural Resources Valorization, Faculty of Sciences, Ibn Zohr University, 80060 Agadir, Morocco; 9 Department of Pharmaceutics, College of Pharmacy, King Saud University, P.O. Box 11451, Riyadh, Saudi Arabia; 10 Laboratory of Therapeutic and Organic Chemistry, Faculty of Pharmacy, University of Montpellier, 34000 Montpellier, France; 11 Department of Biology, Bahir Dar University, P.O. Box 79, Bahir Dar, Ethiopia

Correction to: *Scientific Reports* 10.1038/s41598-024-59577-4, published online 29 April 2024

In the original version of this Article, Md. Rezaul Islam, Md. Shafiqul Islam Sovon, Ummy Amena, Miadur Rahman, Md. Eram Hosen and Ajoy Kumer were incorrectly affiliated with ‘Department of Biology, Bahir Dar University, P.O. Box 79, Bahir Dar, Ethiopia’.

The original Article has been corrected.

