# Supplementary material for: Ligand-based drug design against Herpes Simplex Virus-1 capsid protein by modification of limonene through in silico approaches
Source: Sci Rep. 2024 Apr 29;14:9828. doi: 10.1038/s41598-024-59577-4 (PMC11058824; doi:10.1038/s41598-024-59577-4)

**Supplementary Figure S1: Frontier Molecular Orbitals (HOMO and LUMO) diagram**


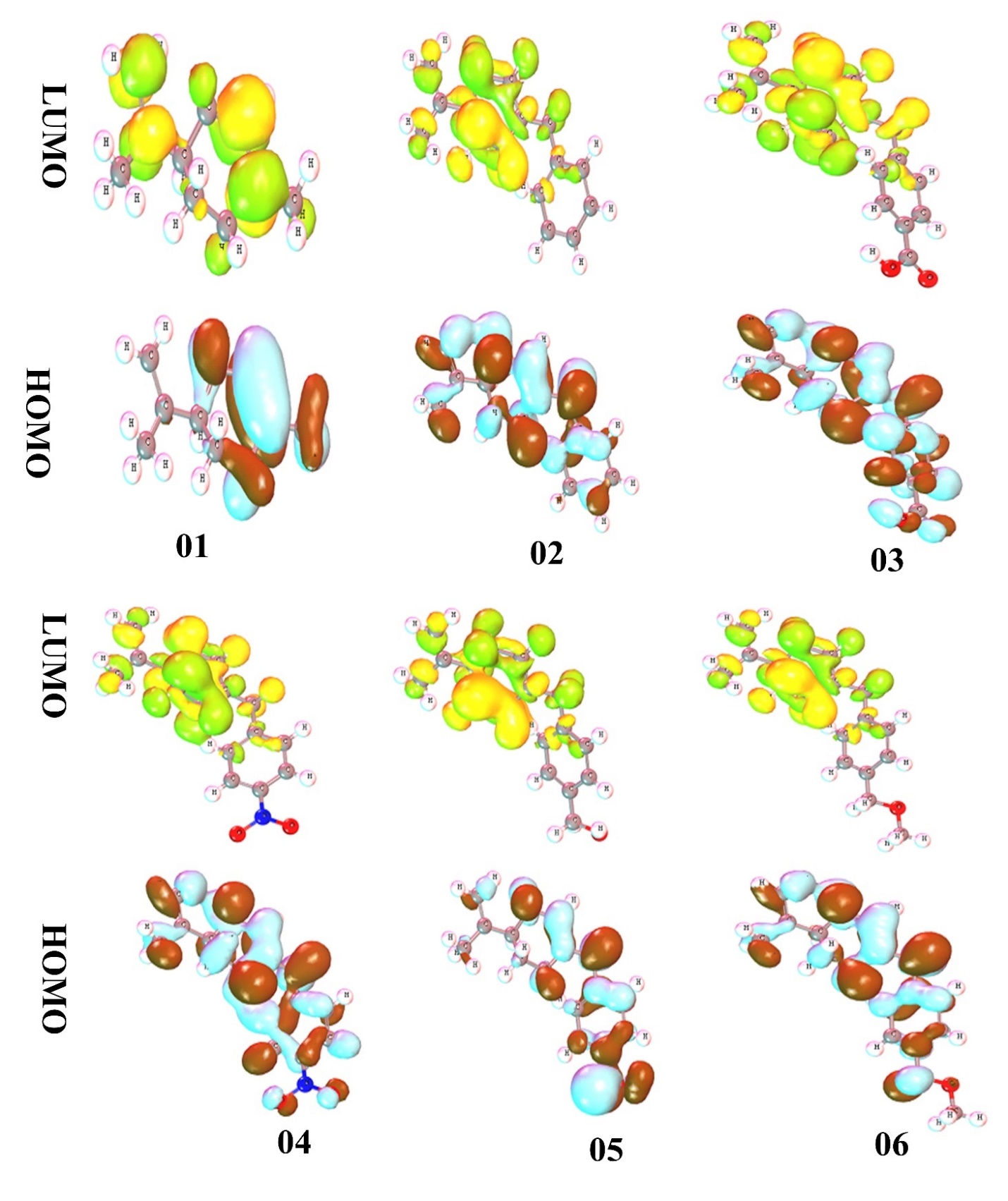


**Supplementary Figure S2:** Molecular electrostatic potential (MEP) mappings.

**
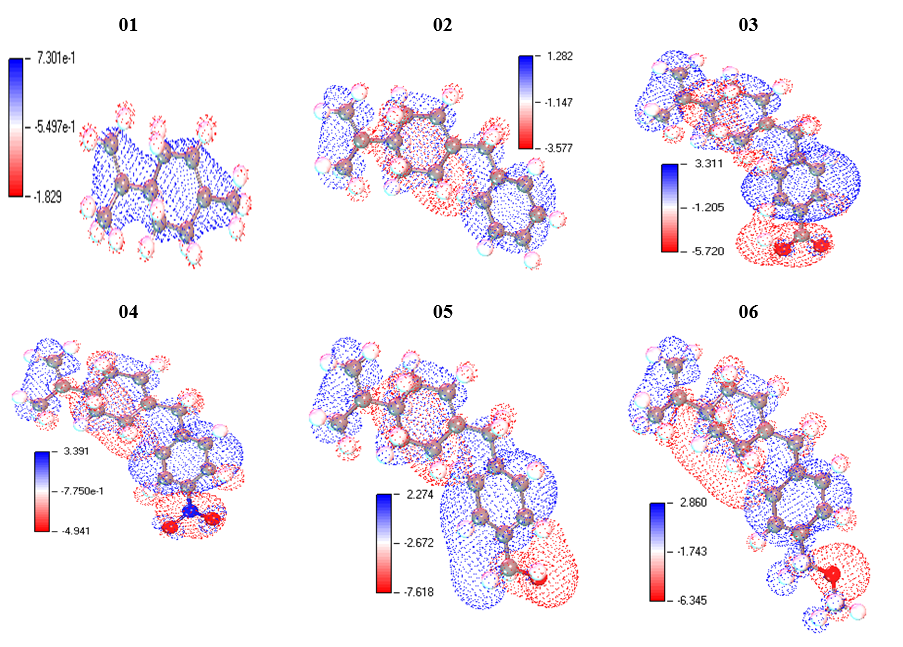
**

**Supplementary Figure S3:** The analysis of MMPBSA analysis binding energy at 100 ns molecular dynamics simulations periods.


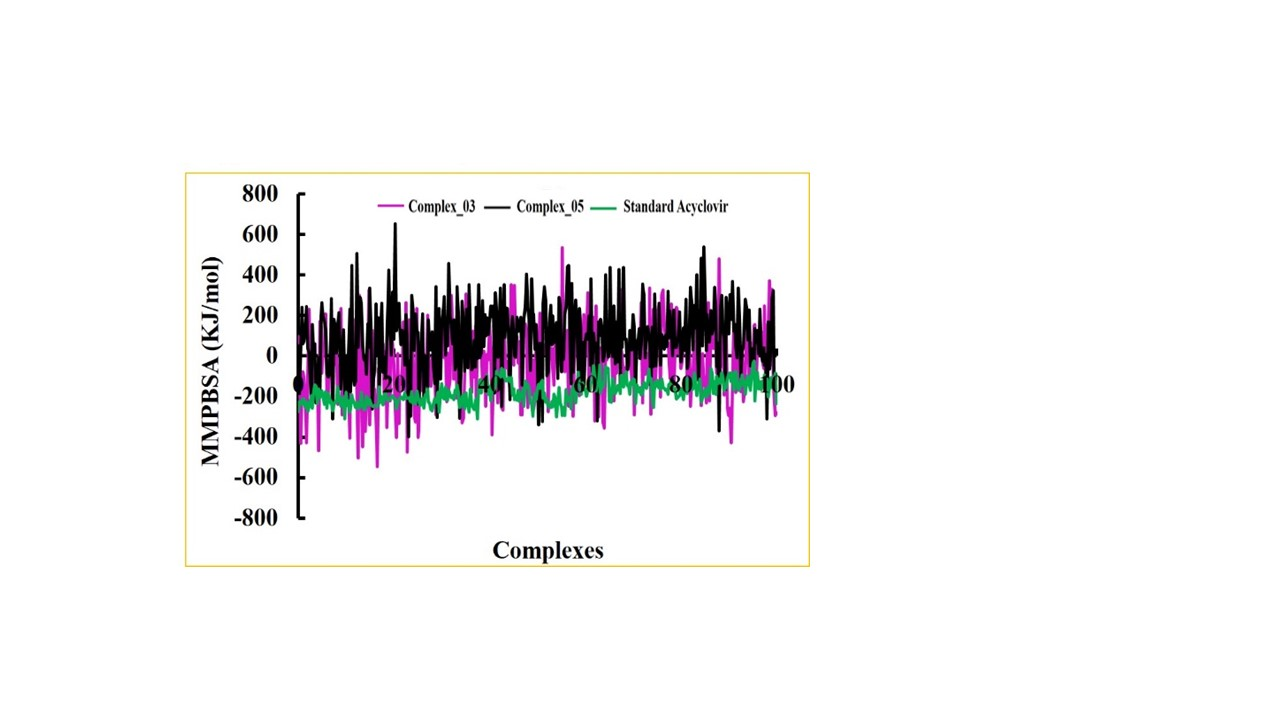

Supplement: Supplementary file 1 — Supplementary Figures. [file 41598_2024_59577_MOESM1_ESM.docx]
